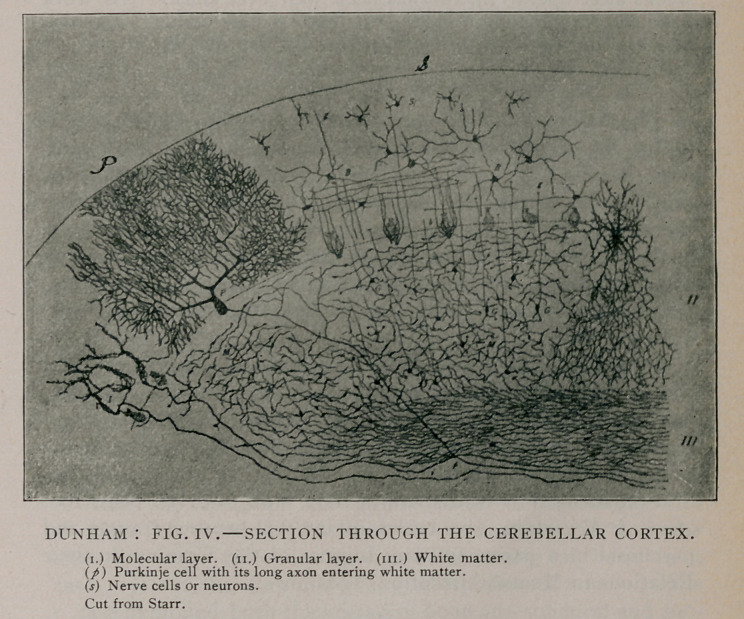# Secondary Traumatic Insanity1Read before section on surgery, Buffalo Academy of Medicine, October 2, 1900; also appeared in competition for the Marcel Hartwig prize for that year.

**Published:** 1902-11

**Authors:** Sydney A. Dunham

**Affiliations:** Buffalo, N. Y.; Superintendent “The Dunham Home,” for selected nervous invalids, 239 Delaware Avenue.


// BUFFALO MEDICAL JOURNAL.

VOL. XLII.—LVIII. NOVEMBER, 1902. NO. 4.

### ORIGINAL COMMUNICATIONS.

### Secondary Traumatic Insanity.[1]

ETIOLOGY, SYMPTOMATOLOGY AND PATHOLOGY, WITH REPORT OF CASE EIGHTEEN YEARS AFTER INJURY WITH RECOVERY AFTER TREPHINING.

BY SYDNEY A. DUNHAM, M. D., Buffalo, N. Y.
Superintendent "The Dunham Home," for selected nervous invalids.

"Canst thou not minister to a mind diseased."—MACBETH.

THE case which I am about to report led me to review the subject of traumatic insanity in recent literature. My impressions and conclusions of this review I present to you with the hope that it will lend interest to the subject. Traumatic insanity has been defined by Theodore Kellogg as mental alienation from damage to the organ of the mind by mechanical violence to the cranium, membranes and cerebral tissue.

He substantiates my conclusion in this case when he says "There are cases where we find intervals of weeks and months or years between the cranial injury, which may have caused slight depression of the skull and the attack of mania or melancholia which may manifest a recurrent tendency."

This patient presented no lesion of the tissues but an exostosis of the button of bone removed at the site of injury, which was the source of cortical irritation. Cases are recorded where slight injuries have become a focus from which extensive alterations develop in the cortex and subjacent part.

Head injuries, as a physical cause of insanity, attract our attention because of the opportunities offered the physician and the hope given the patient. We expect fracture of the skull with depression to give mental disturbances, but there is no doubt that in many cases concussion of the brain without

1. Read before section on surgery, Buffalo Academy of Medicine, October 2, 1900; also appeared in competition for the Marcel Hartwig prize for that year.

fracture has produced or predisposed to insanity. The immediate sensory and motor disturbances after injury are well known, but the psychoses developing months and years after injury to the head are not so well understood but are of greater importance.

William Julius Mickle says traumatism may predispose to, or excite attacks of mental disease—the "spark that happens to fire prepared brain." The symptoms at the time of injury may pass off but leave an impression which leads to instability or leave behind some organic lesion which increases and goes on to mental failure. The traumatic factor may be enhanced by the neurotic or insane diathesis, sensitive, excitable disposition, syphilis or alcoholic excesses.

DUNHAM : FIG. I.—SECONDARY TRAUMATIC INSANITY.

In this case there were no traceable hereditary or acquired neuroses to predispose the patient to insanity, such as instability, insane diathesis, intemperance or syphilis. The initial stadium showed gradual changes in disposition, loss of memory, confused ideas, which later developed into hallucinations, delusions and periodic attacks of mania. The symptoms present were such as headache, vertigo, infidelity in the home, with various disturbances of the special senses.

Exploratory trephining was justified in this case because the operation was free from danger and there was much to be gained if my diagnosis was correct.

PERSONAL AND FAMILY HISTORY.

C. A., born in Germany, aged 42 years; came to this country at the age of 29; always temperate. Father killed by accident. Mother died at the age of 56 of heart disease. One sister living age 37. One sister died at the age of 37; do not know cause.

At the age of 24, patient was hit on top of head by a club in a fight. The next day he felt a soft spot on his head at seat of injury. He was unconscious at the time of the injury about

DUNHAM: FIG. II.—DIAGRAMATIC REPRESENTATION OF A NEURON.

*(Cell body with all its processes.)*

(*a*) Cell body with nucleus and nucleolus.
(*b*) Axon which carries impulses away from cell body.
(*c*) Dendrons ramifying around cell body, conducting impulses to it.

Cut from H. Campbell Thompson's book.

two hours. By pressing on the depressed bone he had pain all through his head. Had no physician at the time. Has never felt good on top of head since injury. Unable to work only half the time for the last two years; does not do work well. Pain in the head over seat of injury or occasional general cranial pain, tinnitus aurium and photopsia. Change of character, such as impatience, apprehension, loss of memory, confusion of ideas, dazed conditions. Physician had recommended asylum treatment one year ago.

*Sensory Symptoms early and constant.*—Sudden sharp noises go to the spot of tenderness. Riding in an elevator would produce fulness in head and cause dizziness. Could not remember anything that was said in a public address. He was irritable and children annoyed him at home. He was unable to saw wood straight and would file the saw the wrong way. He worked at

DUNHAM: FIG. III.—LAYER OF CELLS OF CEREBRAL CORTEX.

(I.) Superficial layer; (II.) Layer of small pyramids; (III.) Large pyramids; (IV.) Deep layer; (V.) White matter containing neurons from pyramidal cells.

Cut from M. Allen Starr's book.

washing dishes in a bakery, and although the dishes were chiefly tin, his work was inaccurate and not very satisfactory. That was all he was able to do of late years. Could not take alcoholic stimulants without having a headache and losing himself.

*Physical Symptoms.*—Tenderness always present at seat of injury. Pressure at that point very painful. Sense of weight and pressure always on top of head. Occasional shooting pain radiating from seat of injury. Anything that increased intra-cranial pressure would aggravate his trouble.

*Mental and Moral Symptoms.*—In the last two years has been getting worse and has had several attacks of mania, followed sometimes by great mental depression. He was suicidal. These attacks were periodical and getting more frequent and prolonged. He was changed in character, despondent, impatient and apprehensive. There was loss of memory and confusion of ideas.

DUNHAM: FIG. IV.—SECTION THROUGH THE CEREBELLAR CORTEX.

(I.) Molecular layer. (II.) Granular layer. (III.) White matter.
(*p*) Purkinje cell with its long axon entering white matter.
(*s*) Nerve cells or neurons.

Cut from Starr.

Patient entered Riverside Hospital, December 1, after one of his periodical mental storms. Before operation his pulse was under 70; temperature, below 98°. This has been characteristic of his physical suboxygenation, or as we would say, lowered vitality. A button of bone was removed the following day by Dr. William R. Henderson, of Buffalo. The depressed bone removed was one inch in diameter and situated over the antero-superior angle of parietal bone on right side. It showed that inner table of skull had been fractured, and thickening of inner side of bone had occurred. Dura little thickened but no adhesions. Pulsation fair, hence dura was not opened.

Day after operation temperature and pulse normal. Patient said his head felt much lighter and clearer, and pressure was entirely gone. His expression was much brighter and he was happy all through.

Left the hospital on tenth day feeling well. Reaching home he went to sawing wood with his head down, also hit his head against beam in woodshed. This was followed by hot head and delirium for two days, but was at once controlled by ice cap, bromides and quiet in bed.

He resumed his work in the bakery on January 29, 1901, eight weeks after operation, and has been well ever since. His work has been satisfactory. He is practically a new man. He is still at work and there is no return of head symptoms.

### PATHOLOGY OF TRAUMATIC INSANITY.

The pathology of traumatic insanity may be briefly given as follows: reveals dural and cortical adhesions as the most common lesions. We may find thickening of the membranes or bones, inflammatory and degenerative changes extending from the seat of injury; lesions opposite side of injury may occur by contracoup, *e.g.*, (hemorrhage resulting in meningitis and areas of adhesions) osteophytes or splinters puncturing dura and brain, from inner table of skull, substructural or subcortical cysts, necrosed bone.

When secondary traumatic insanity occurs sometime after the injury, when the interval is considerable, the question naturally arises, was the injury the cause? Cases have been reported in which insanity has been ascribed to the injury twenty-three years before.

Statistics by J. Christian, in 1899, Vol. XVIII., page 198, *Arch. de Neurologie*, give tables of interval between trauma and psychosis in a paper entitled, Injuries of the Cranium in their Relations to Mental Alienation, as follows:

| Interval. | Insanity. | General Paralysis. | Dementia. | Epilepsy. | Total. |
|---|---|---|---|---|---|
| 1 to 5 years | 17 | 23 | 8 | 6 | 54 |
| 5 to 10 years | 6 | 7 | 4 | 4 | 21 |
| 10 to 20 years | 3 | 4 | 2 | 2 | 11 |
| 20 to 30 years | 2 | 4 | 1 | .. | 7 |
| Over 30 years | 1 | 5 | 1 | .. | 7 |
| | 29 | 43 | 16 | 12 | 100 |

### STOLPER'S STATISTICS.

He has gone over personally the records of a large hospital for ten years, 1886 to 1896, inclusive. There were 961 injuries of the head. There were 12 which were followed by insanity,

1.2 per cent. Of the 12 cases, 1 only was a comparatively mild injury, all the others being severe fractures of the dome or base of the skull. As the total number of severe injuries of this sort was 138, it follows that the 11 cases of insanity following severe trauma amounts to 8 per cent.

He found out of a dozen or more insane asylums, records representing 18,606 insane, there were 480 cases, 2.5 per cent. of traumatic insanity.

POWELL'S AND HARRISON'S STATISTICS.

The surgical treatment of traumatic insanity, with a contribution of three successful operations, by James Harrison, Liverpool, *Med. Cir. Jour.*, 1898, XVIII., p. 243.

Dr. Herbert A. Powell collected all the cases in literature from 1878 to 1890, and published them in a monograph on The Surgical Aspects of Chronic Insanity. Powell found records of 67 cases and analysed them. Harrison took up the literature where Powell left it off, and could find but 10 additional cases (including his own three), to date (1898). Of this number only 7 had been operated upon at the time of the accident. Forty-eight per cent. of the 70 cases showed depression of the skull which could be felt.

Of cases in which there was no depression, there were cicatrices, evidences of old contusions, tender points, and the like, as well as trephine openings, *i.e.*, when the operation was actually performed these symptoms furnished the indications for intervention.

After the operation the lesions found in association with 48 cases of depressed fracture were osteophytes or splinters from the inner table in 13 cases, thickening of bone in 9 cases, cysts in the dura mater in 3 cases, diseased bone 1, while 1 case exhibited both thickening of bone and subdural and sub-c ortical cysts, and there was 1 case of a bullet lodged in the dura.

In 20 cases in which there were only cicatrices or tender points, there were thickened bone in 6 cases, osteophytes and splinters in 2 cases, serous cyst in 1 case, and adhesion between the brain and dura in 1 case; while in 8 cases nothing was found, and 6 of the 8 made good recoveries.

Taking cases as a whole (77) the dura was said to have been adhesive in 14, and the pericranium in 2.

*Results of Operation.*—Of the 77 cases, 5 deaths occurred, and of 57 reported in the last 17 years, only 2 died. With regard to mental state, 51 of the 77 recovered and 12 were much benefited; 5 improved slightly, and 4 not at all.

I have selected the following report of cases from literature on the subject, because they correspond most closely in clinical history and mode of treatment to that of my own case.

C. LOCKHART ROBERTSON'S CASE.

On application of the trephine to the Treatment of Insanity, the Result of Injury to the Head, *London Journal of Psychological Medicine*, Vol. I., 1848, p. 155.

Patient aged 23, sailor; first seen 1845. Ten years earlier he fell from a mast and the accident was followed by acute mania. In six weeks he regained the use of his faculties, but his character changed for the worse. He was passionate and formed intense dislikes, and for this reason was incarcerated in an asylum. Under medical treatment he improved somewhat, but not enough to gain his freedom. About this time he was seen by Robertson who found a distinct depression at the posterior superior margin of the parietal bone, to which locality he referred his pains.

Trephining was decided upon and was performed by Surgeon Furnes. The dura was very adherent at this point. There was no line of fracture in the bone, only a bend. In about 10 or 11 weeks later, patient was discharged perfectly cured.

FLETCHER'S CASES.

In the *American Journal of Insanity*, Vol. 44, W. B., 1887-88, Fletcher, Superintendent Indiana State Hospital for the Insane, reports 8 cases of trephining for secondary traumatic insanity, 4 of them I find similar to my own.

Case I.—Male, age 35, was injured at the age of 29 by falling from a scaffold. Depression of bone at osculation, right parietal and occipital; lost memory and had become inebriate, also had epileptic convulsions. Discharged free from pain and mental disturbances after trephining. An interval of six years.

Case II.—Male, age 47, injured at the age of 44 by being hit on the head by a stove lid. Patient became unconscious for six hours; in bed several days. Resumed work for six months and gave up. He was negligent about clothing and was suicidal and melancholy. Small scar, size of grape seed over parietal suture, inch and one-half from the coronal, scalp adherent and very slight depression of the skull. Trephine was one inch long and a half inch wide; spicula of bone of inner tablet punctured the dura; went home on the seventh day a new man, as he said. An interval of three years.

Case III.—Male, age 37, injury at the age of 13 by small wagon wheel. Epilepsy at 20 and in a fit of frenzy killed his 2-year-old child at 24. Was violent, homicidal, several violent convulsions a month, no depression at the seat of injury. The trephine of bone was one and a half inch in length and one inch in width. Convulsions lessened and at last report had had no convulsions for six months. An interval of 24 years.

Case IV.—Male, age 27, injured at 25, destructive and suicidal; constant pain in the region of the parietal frontal suture, scalp wound very plain, no fracture. Patient benefited by trephining, but still has pain. An interval of two years.

BACON'S CASE.

Trephining of the skull in the case of a lunatic nineteen months after the receipt of a blow on the head. Complete recovery. G. Mackenzie Bacon, *British Journal Mental Science*, 1880-81, XXVI., p. 551.

Male, age 38. Previous health always good but nervous and slender. Was a carpenter; habits good.

In August, 1878, while at work a hammer fell from a height of six feet, striking the top of his head. He was not stunned nor did any untoward symptoms develop, but he "never felt the same" after the blow. Earliest sensations similar to "cold in the head." In the following January he took to his bed where he remained for many weeks—symptoms not stated. When he recovered he was unable to work, as attempts to this end produced sensations of vertigo and numbness in the legs. Could not apply his mind.

In October, 1879, he sought hospital treatment. The principal symptom was a "scrunching" feeling in the vertex, referable to the stellate scar which marked the site of the injury. Disposition irritable and morose and talked of suicide. Other symptoms were dragging pains at the vertex, without intermission; aching pains in both arms and along the inside of both legs; noises in the ears and inequality in the size of the pupils. Very little sleep.

On January 1, 1880, a determined attempt at suicide; he fell fifteen feet down a stairway, spraining an ankle. Transferred from hospital to insane asylum. Was unable to control sudden impulses, restless and sleepless. A slight depression of skull on vertex (left parietal bone). After a month's care was better, but could not control thoughts or acts. Memory poor for recent events.

On March 12, 1880, a year and seven months after injury, it was decided to trephine; operation performed by Wherry, of Cambridge. A piece of parietal bone was taken out at the seat of injury. The dura was purplish in hue, but sound and bulged with pulsations, into the wound. The trephine button was three quarters of an inch in diameter, and there had been no fracture. The wound healed rapidly and well.

On March 30, his mind was in a much improved state. It was thought best for him to return to his work by June. He was found to be fully able to earn his living.

### CAUSES.

The causes which lead up to such severe mental disturbances after so slight an injury with such a long interval, are molecular changes in the neuron, produced by disturbed circulation and impaired nutrition (Fig. iv.) When we think of the minute arteriole twigs passing at right angles into the cortex from pia mater, which have to do with nutrition, we can better understand how an embolism or thrombus, or any foreign substance producing pressure may affect the gray matter and the subjacent cells. The lymph spaces between the arachnoid and pia mater and the lymph channels around the blood vessels, which are so common in the brain, help us to understand how we get cerebral changes when the circulation of either blood or lymph is obstructed. Slight pressure may also interfere with the pulsatory movement of the brain. The functions of the neurons are normally carried on when their nutrition is perfect. The physiological rhythm of the ganglion cells, such as repose and activity, is disturbed when their nutrition is impaired.

Brain pressure is constantly attended by disturbances of the circulation and nutrition of the brain. Meynert concludes that all stimuli acting on the brain (sensorium) create vascular movements and disturb the periodic changes in the condition of the vessels. If the pressure is moderate, the symptoms may remain latent or only show themselves as headache, vertigo, weakness or disturbances of sensory functions.

Recent discoveries in the structure and functions of the nerves help us to explain fully these latent mental manifestations in brain pressure. (Fig. iii.)

The dynamic energy of the neurons is weakened by slight pressure on the ganglion cells, and indirectly by disturbances of circulation of blood and lymph in the subjacent parts. All neurons are contiguous but not continuous with other neurons. Each neuron is a unit anatomically and physiologically (Fig. ii.) The extending and retracting of the processes of the neurons, like that of the devil fish, are lessened when under continued pressure. The more frequent the various processes of the nerves are associated, the stronger they become. Failure to remember recent events is due to the fact that the associations of the axon are not well made. The weaker the neuron in energy, the more difficult to recall what has transpired; in other words, memory is poor and getting worse.

My case gives a good picture of the failing energy of the nerve centers. This condition in time would have probably terminated in degeneration of nerve cells.

The most important fact in physiology of the nervous system is the independence of the neuron. Clinical observations make it certain that the cerebral cortex is the physical basis of the mind. Certain attributes of the mind are disturbed by certain diseased areas as well as the paralysis of sensation and motion, which always follows injury to certain portions of the cortex.

BRIEF SUMMARY.

Do not underestimate slightly depressed fracture of the skull, for grave symptoms may develop many years afterward.

Whatever increases intracranial pressure aggravates the trouble, hence the inability of these patients to use stimulants.

The symptoms of general paresis, which may develop sometime after injury to cranium, may be relieved by trephining.

Depressed fractures of vertex of head are followed by disturbances of memory, judgment and will power.

Old cases are destined to mania and paresis.

239 DELAWARE AVENUE.

## Figures and Tables

**Fig. I. f1:**
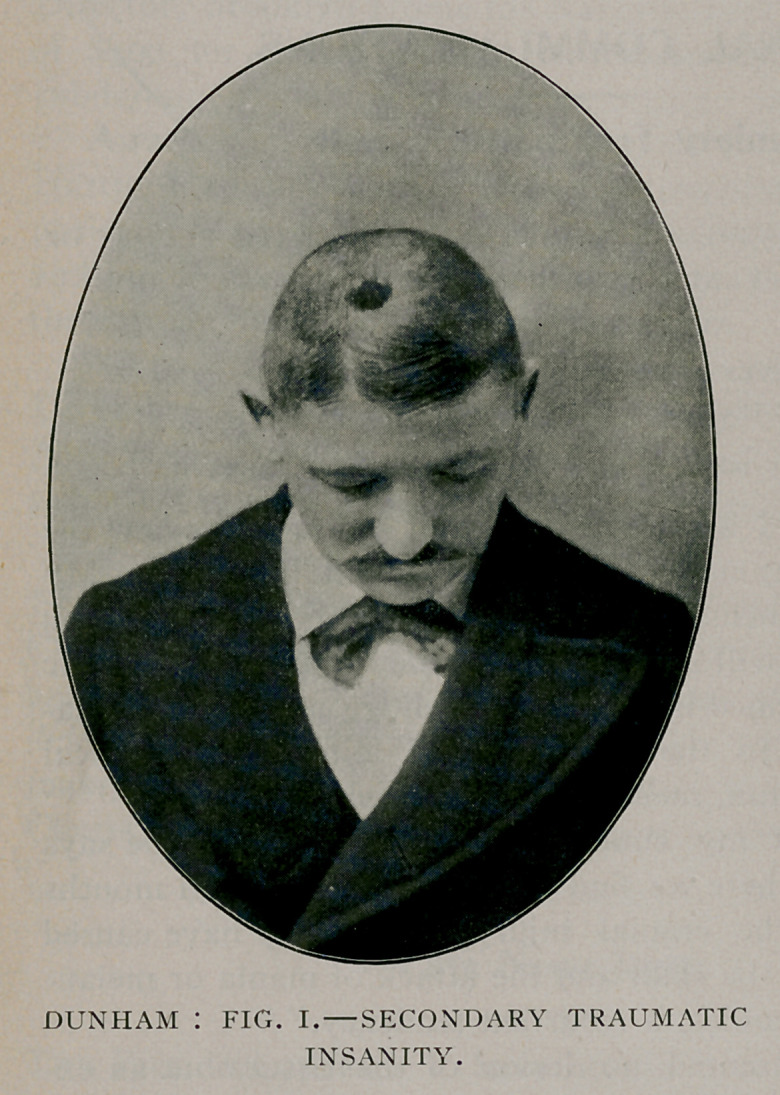


**Fig. II. f2:**
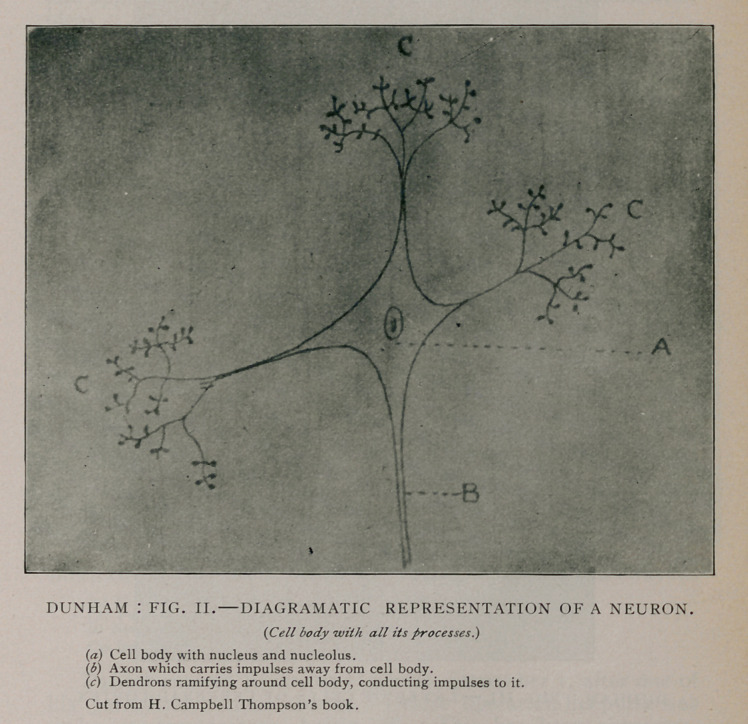


**Fig. III. f3:**
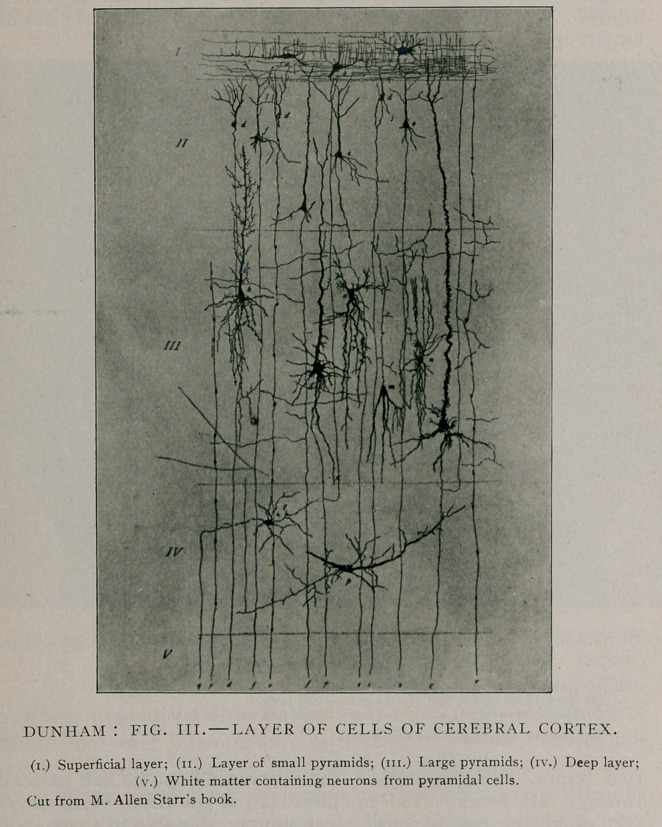


**Fig. IV. f4:**